# Validation of a machine learning algorithm to identify pulmonary vein isolation during ablation procedures for the treatment of atrial fibrillation: results of the PVISION study

**DOI:** 10.1093/europace/euae116

**Published:** 2024-04-29

**Authors:** Jan De Pooter, Liesbeth Timmers, Serge Boveda, Stephane Combes, Sebastien Knecht, Alexandre Almorad, Carlos De Asmundis, Mattias Duytschaever

**Affiliations:** Heart Center, UZ Ghent, Corneel Heymanslaan 10, 9000 Ghent, Belgium; Heart Center, UZ Ghent, Corneel Heymanslaan 10, 9000 Ghent, Belgium; Clinique Pasteur, Toulouse, France; Clinique Pasteur, Toulouse, France; AZ Sint-Jan, Brugge, Belgium; UZ Brussel, Brussels, Belgium; UZ Brussel, Brussels, Belgium; AZ Sint-Jan, Brugge, Belgium

**Keywords:** Atrial fibrillation, Pulmonary vein isolation, Machine learning

## Abstract

**Aims:**

Pulmonary vein isolation (PVI) is the cornerstone of ablation for atrial fibrillation. Confirmation of PVI can be challenging due to the presence of far-field electrograms (EGMs) and sometimes requires additional pacing manoeuvres or mapping. This prospective multicentre study assessed the agreement between a previously trained automated algorithm designed to determine vein isolation status with expert opinion in a real-world clinical setting.

**Methods and results:**

Consecutive patients scheduled for PVI were recruited at four centres. The ECGenius electrophysiology (EP) recording system (CathVision ApS, Copenhagen, Denmark) was connected in parallel with the existing system in the laboratory. Electrograms from a circular mapping catheter were annotated during sinus rhythm at baseline pre-ablation, time of isolation, and post-ablation. The ground truth for isolation status was based on operator opinion. The algorithm was applied to the collected PV signals off-line and compared with expert opinion. The primary endpoint was a sensitivity and specificity exceeding 80%. Overall, 498 EGMs (248 at baseline and 250 at PVI) with 5473 individual PV beats from 89 patients (32 females, 62 ± 12 years) were analysed. The algorithm performance reached an area under the curve (AUC) of 92% and met the primary study endpoint with a sensitivity and specificity of 86 and 87%, respectively (*P* = 0.005; *P* = 0.004). The algorithm had an accuracy rate of 87% in classifying the time of isolation.

**Conclusion:**

This study validated an automated algorithm using machine learning to assess the isolation status of pulmonary veins in patients undergoing PVI with different ablation modalities. The algorithm reached an AUC of 92%, with both sensitivity and specificity exceeding the primary study endpoints.

## Graphical Abstract

**Graphical Abstract:**
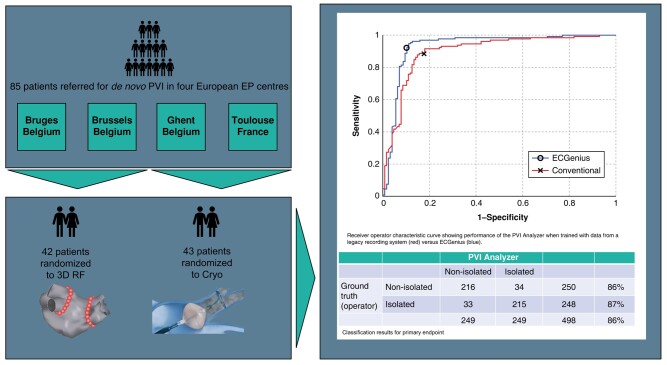

**What’s new?**

- Evaluation of a previously trained and tested machine learning (ML) algorithm designed to assess pulmonary vein (PV) status during PV isolation procedures in a real-world clinical setting showed an accuracy rate of 86% when compared with operator opinion.
- An ML algorithm trained on four PV cardiac electrogram (EGM) features achieved an area under the curve of 92%.
- Machine learning applied to cardiac EGMs may provide an additional useful diagnostic tool to assist vein isolation status.

## Introduction

Complete electrical isolation of the pulmonary veins (PVs) has become the cornerstone of treatment for paroxysmal atrial fibrillation (AF).^1^ A number of techniques and energy sources can be used to achieve PV isolation (PVI), including point-by-point radiofrequency (RF) energy,^2^ cryoballoon (CB),^3^ and more recently, pulsed field ablation (PFA).^4^ Both RF and a CB may use a circular mapping catheter (CMC) positioned in the PV of interest to confirm the complete abolition of PV potentials. However, classification of PV status as either isolated or non-isolated can be made challenging by the presence of far-field components that create complex bipolar electrograms (EGMs). These far-field signals originate from adjacent anatomical structures such as the left atrial appendage,^5^ superior caval vein,^6^ vein of Marshall,^7^ ipsilateral veins, or the left atrium.

While pacing manoeuvres and other techniques may help in the discrimination of far- and near-field components of CMC signals, it has been noted that the results of multi-centre clinical trials for PVI have shown large differences in outcomes between centres,^8^ which may be attributed to inter-operator variability as a result of individual operator experience and centre volume. It has also been shown that inter-observer variability can occur even in the most reputed centres when PV potentials exhibit complex morphologies.^9^ Indeed, in the original paper describing the PVI Analyzer algorithm, a study was performed to examine the accuracy of physician classification of PV status compared with a gold standard of status defined by non-conducted automaticity, with the overall rate of accuracy of six experienced electrophysiologists being 78%.^10^ However, in a recent study testing an algorithm designed to detect far-field signals, five experienced operators achieved an intraclass correlation of only 0.69 when classifying near- and far-field PV potentials.^11^

A machine learning (ML) model, based on a support vector machine (SVM) classification using signal processing techniques to classify a range of PV potential types recorded on an 8- or 10-pole CMC, was trained and tested as previously described.^10,12^ In this ML training data set, ground truth for isolation was determined using only EGMs from veins showing non-conducted automaticity (*n* = 1440). After training, this algorithm achieved an overall accuracy rate of 93% during initial validation.^10^

The current study presents the results of the PVISION multi-centre trial to test the PVI Analyzer algorithm designed to classify PVs as either isolated or non-isolated against expert opinion in a real-world clinical setting.

## Methods

**Figure 1:**
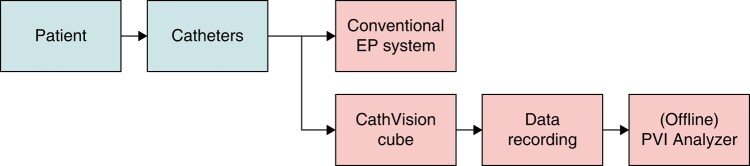
The signal pathway from the patient through the CathVision ECGenius to the offline PVI Analyzer.

The PVISION study was designed as a real-world validation of a previously trained and tested ML algorithm to test the agreement between an experienced operator and the algorithm. The aim of the study was to determine the sensitivity and specificity of the ECGenius® EP recording system’s PVI Analyzer software algorithm (CathVision ApS, Copenhagen, Denmark) to detect PVI in patients undergoing ablation using a CB or a three-dimensional (3D) navigation-assisted RF. Procedures performed with the CB used the Arctic Front Advance system with Achieve CMC (Medtronic, Inc.) system and those performed with RF used the CARTO 3D navigation system, Navistar ablation catheter, and 10-pole Lasso CMC (Biosense Webster, Irvine, CA, USA). For the RF procedures, power settings followed either the CLOSE-PVI protocol (35–40 W, ablation index–guided 450 for the anterior wall and 350 for the posterior wall) or a high-power short-duration approach (90 W, 4 s). As the algorithm is agnostic to the ablation protocol, this was not mandated in the study protocol, and therefore, data pertaining to the approach used by individual operators were not collected during the procedure. Patients were recruited at four centres in France and Belgium (Clinique Pasteur, Toulouse, France; Universitair Ziekenhuis, Ghent, Belgium; Algemeen Ziekenhuis, Sint-Jan Brugge, Belgium; and Universitair Ziekenhuis, Brussels, Belgium). A novel EP recording system was connected in parallel with the laboratory’s existing recording and mapping systems (*Figure 1*).

Simultaneous 10 s EGM recordings of CMC EGMs from the laboratory’s existing system and the ECGenius System were recorded and annotated in the event log of both recording systems during sinus rhythm (SR) at baseline prior to ablation and following the last ablation when the operator had documented that veins were electrically isolated. In each case, the ablation procedure was performed according to the standard of care at the investigational site. Pulmonary vein isolation status was based on expert operator interpretation that (i) PV signals were completely abolished, (ii) conduction of spontaneous PV ectopy/automaticity to the left atrium (LA) was abolished, or (iii) documentation by pacing that conduction block had been established between the left atrium and the PV.

The ethics committee of each site approved the protocol, and the investigator obtained written informed consent from each patient prior to enrolment and participation in the study. The study was registered on the public database clinicaltrials.org with the identifier NCT05043883 and conducted in accordance with the Declaration of Helsinki.

### Investigational device

The ECGenius System is an EP recording system that records and displays cardiac electrical signals. The main hardware amplifier of the ECGenius System, the ‘CathVision Cube’, acquires and digitizes signals from third-party catheters and sensors connected to patients. It sends the signals to the system software, providing the ability to record, display, and interactively analyse them.

**Figure 2:**
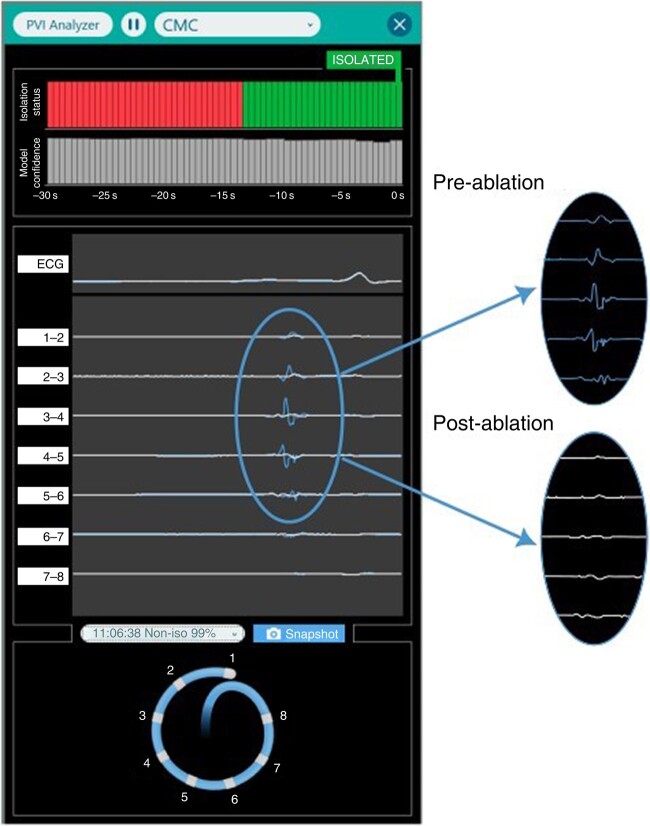
A user interface of the PVI Analyzer showing an avatar of the CMC, PV EGMs, and classification with algorithm confidence. The blue PV electrograms are a snapshot of the pre-isolation signals superimposed on the current signals. CMC, circular mapping catheter; PV, pulmonary vein.

As a software module within the ECGenius System, the PVI Analyzer application is intended for use during a catheter-based PVI procedure to support the decision that a vein has indeed been electrically isolated (*Figure 2*). The PVI Analyzer model computes an isolation index every 500 ms, which is a number between 0 and 100 that indicates the assessment of isolation for each beat. The ML model used for the PVI Analyzer employs an SVM algorithm trained on four EGM features. These features were extracted by creating a 128 ms window of interest around the potential PV EGM. Within this window, the four most dominant local peaks or valleys were identified. The parameters used to define a peak/valley were an absolute amplitude >0.025 mV. These local extrema were used to compute the following scalar features: (i) typology: ‘Low voltage’, if there was no measurable peak; ‘Monophasic’, if there was a single peak; ‘Biphasic’, if there were two peaks; ‘Triphasic’, if there were three; ‘Multiphasic’, if there were four peaks; and ‘Double potential’, if there were at least two peaks with a minimum distance of 25 ms between them; (ii) peak-to-peak amplitude defined as the difference in amplitude between the highest peak and the lowest valley; (iii) maximum d*v*/d*t*: the d*v*/d*t* of the potentials around the peaks and valleys are calculated, and the maximum absolute d*v*/d*t* is computed; (iv) sharpest angle: the minimal angle between the upstroke and the downstroke of the peak or valley.

### Data analysis

In the PVISION study, the PVI Analyzer software was installed on a separate system to perform the calculations retrospectively and off-line. Ten second EGM recordings were collected pre-ablation and post-ablation during the ablation procedure. All EP signals from the procedure were exported to an external data storage device following the procedure and used later to analyse and classify pre-ablation and post-ablation recordings as PV isolated or PV non-isolated. For ensuring consistency across EGMs, only the last computed isolation index per EGM recording was used to compute the primary endpoints, and other isolation indices were disregarded. An index threshold of 50 was used for the isolation index for each EGM recording, with values below 50 classified as non-isolated, while values at or above 50 were considered to be isolated (at a threshold of 50, the impact/cost of a false positive or a false negative are considered identical). Measurements were excluded from the analysis if the PVI Analyzer did not return a value due to high levels of noise, power interferences, or other artefacts. Computation of the primary endpoint required the EGM recordings from stable baseline (pre-ablation) and isolation (post-ablation) for each PV. The operating clinicians were not informed of the results of the PVI Analyzer in individual cases until data from all cases were collected and analysed. Whenever possible during the procedure, the physician marked in the ECGenius event log the earliest beat at which a vein could be confirmed isolated. Electrograms from such registrations were analysed with the PVI Analyzer to compute the accuracy of the isolation classification at the time of isolation.

### Study inclusion/exclusion criteria

The study included patients who were undergoing first-time PVI for the treatment of AF; these patients were either males or non-pregnant or nursing females, aged ≥21 years of age and were able and willing to provide written informed consent. Only PVs with interpretable 10 s EGM recordings at baseline and following PVI were included in the study analysis.

The exclusion criteria included:

- Patients who were pregnant or nursing females.
- Patients participating in a concurrent investigational drug or device study that interfered with the PVISION study.
- Patients who had a prior ablation procedure.
- Patients with other anatomic or comorbid conditions, or other medical, social, or psychological conditions that, in the investigator’s opinion, could limit the ability of patients to participate in the clinical investigation or to comply with follow-up requirements, or impact the scientific soundness of the clinical investigation results.
- Patients with a life expectancy <12 months.
- Patients who, in the opinion of the investigator, are considered part of any vulnerable population.

### Primary performance endpoint

The primary performance endpoint was the sensitivity and specificity of the PVI Analyzer software using the ECGenius Recording System for PVI following ablation. Expert assessment by the operator using aforementioned criteria that a PV was isolated served as the ground truth to which the PVI Analyzer determination was compared. The lower limit performance criteria were, based on a review of the published literature,^10,12^ prespecified at >80% for both sensitivity and specificity.

### Secondary performance endpoints

Secondary performance endpoints, defined to determine the feasibility of assessing the performance of the PVI Analyzer software in real time to guide ablation, included:

- Accuracy of automated PVI Analyzer classification of PVI in SR during PVI CB ablation with a performance goal rate superior to 80%.
- Accuracy of automated PVI Analyzer classification of PVI in SR during RF ablation with a performance goal rate superior to 80%.
- Feasibility of continuous ‘real-time’ PVI Analyzer analysis during PVI ablation treatment:

Test–retest accuracy of the PVI Analyzer classification comparing non-overlapping beats selected from within each EGM. The retest accuracy is computed as the proportional agreement between the two last non-overlapping samples within an EGM, compared with a performance goal rate superior to 90%, considered sufficient for a real-time assessment.

- Performance assessment of PVI Analyzer classification accuracy at the time of expert-defined isolation before the end of the PVI ablation procedure:

This secondary endpoint aims to determine the sensitivity of the PVI Analyzer at the time of isolation, which requires the EGMs at the expert-defined time of isolation during the procedure. Annotation at the time of isolation required direct observation of isolation, and this was recorded for 116 (34%) valid EGMs. During RF procedures, the time of isolation can be challenging to observe, and during CB application, freeze noise from the CB can make identification of the time of isolation difficult. Each annotated EGM is processed by the PVI Analyzer and a single result from each EGM is used for assessment.

- Comparison of algorithm performance on the same data recorded by CathVision ECGenius and Boston Scientific LSPro systems.

This secondary endpoint focuses on the comparison between PVI Analyzer performance from data collected from the ECGenius System and data from the conventional EP system. Data from the conventional recording systems were received from the centres and processed. LSPro data were exported manually as anonymized ASCII files using the LSPro software. All relevant pages were exported. The ASCII files could be read in, and processed by, the PVI Analyzer.

### Statistical analysis

#### Hypotheses testing

The study was designed as a one-sample superiority test of the sampled outcome specificity and sensitivity independently. The success of the PVI Analyzer output was assumed to follow a binominal distribution with a given true proportion of 87.50%. This value was based on the published performance of earlier versions of the algorithm and the adaptation of the algorithm to the final device. According to the primary endpoints, the study tested whether both specificity and sensitivity were superior to 80%.

#### Sample size justification

With a lower bound of 95% confidence interval (CI; *α* = 0.05) and at a statistical power of 95% (*β* = 0.05), a sample size of 210 EGMs at both T-baseline (specificity) and T-final (sensitivity) was required. For the primary endpoint, it was required that EGMs were recorded in SR and with a verified isolation status at T-final, according to the predefined criteria. A non-SR (NSR) occurrence rate of up to 35% was expected at baseline, with a 10% dropout of verified isolation labels and other deviations. Taking this into consideration, a total of 360 EGMs were required. For each subject, up to four PV EGMs could be obtained, which were assumed to be independent samples. Thus, a total of 90 subjects were expected to be required to reach the sample size requisites for both specificity and sensitivity.

#### Device performance analysis

As the PVI Analyzer software classifies an EGM as a binary outcome, either positive (isolated) or negative (non-isolated), the performance was evaluated separately for specificity and sensitivity. To be able to examine a correct classification, the results obtained from the analysis of the PVI Analyzer were compared with the outcomes from the conventional system used at each site per standard of care. In this context, EGMs recorded at ‘T-baseline’ (prior to isolation) were labelled as ground-truth negative (non-isolated), and EGMs recorded at ‘T-final’ (after isolation) were labelled as ground-truth positive (isolated) when they conformed to at least one of the criteria. The primary performance endpoint results are described as the percentage and 95% CI of sensitivity and specificity. A *z*-test was used for the superiority test of the primary performance endpoint. A one-tailed test with a significance level of 95% (*α* = 0.05) was applied. Additionally, secondary performance endpoints were analysed to determine the feasibility of time-critical assessment of PVI analysis and rhythm-dependent performance using the PVI Analyzer software with the CathVision ECGenius System. The different statistical analyses applied for each endpoint are described as follows:

- Automated PVI Analyzer classification of PVI in SR from the EP procedure until study completion at discharge (an average of 24 h), with a specificity and sensitivity superior to 80%. A superiority *z*-test for specificity and sensitivity was performed.
- Feasibility of ‘real-time’ assessment of the PVI Analyzer during PVI ablation treatment. Retest the accuracy of PVI Analyzer classification of isolation status at different time points before, during, and after ablation. Accuracy is defined as the pairwise proportionate agreement of PVI Analyzer re-classification of pairs of non-overlapping samples within 10 s before, during, and after ablation. A *z*-test for accuracy was performed.
- PVI Analyzer classification accuracy at the time of expert-defined isolation. For this specific endpoint, a *z*-test for sensitivity was performed to assess the sensitivity of the PVI Analyzer analysis to identify expert-defined isolation during the PVI ablation procedure.
- Comparison of device performance with the conventional system from the EP procedure until study completion at discharge (an average of 24 h). In this case, classification outcome (specificity and sensitivity of the PVI Analyzer) was compared with the classifications from the conventional system.

The results were summarized using descriptive summary statistics, and a paired McNemar’s *χ*^2^ test was performed to analyse the differences in classification performances. Finally, as the sample size was not adjusted to detect the differences for variables other than the primary endpoint, the *P*-values obtained from these additional analyses were found to be complementary to the primary endpoint results and were only for the purpose of hypothesis generation.

## Results

A total of 92 patients were screened to participate in the clinical study. Patients were enrolled at the four investigational sites between 15 September 2021 and 22 June 2022 (see *Table 1* for details). Three patients were considered screening failures since they did not meet all eligibility criteria and were therefore excluded from the study. As such, the clinical study successfully enrolled a total of 89 patients. Clinical characteristics and demographic information of the 89 patients are presented in *Table 2*. The patients were adults with a mean age of 62.69 ± 12.26 years, and 37% of participants were female. The mean time from AF diagnosis to ablation was 23.98 ± 55.31 months. With regard to the type of AF presented by the patients, paroxysmal AF was the most frequent, reported in 81% of the cases, while 13% of the subjects had persistent AF (the AF type of five patients was not reported).

**Table 1:** Patient enrolment by clinical site

Site name	Country	Subjects successfully enrolled	Subjects with screening failures	Subjects with procedural deviations	Subjects in analysis population after EGM exclusion
Clinique Pasteur Toulouse	France	12 (13%)	1 (33%)	0 (0%)	10 (13%)
UZ Ghent	Belgium	24 (27%)	0 (0%)	2 (50%)	20 (27%)
AZ Sint-Jan Brugge	Belgium	25 (28%)	1 (33%)	2 (50%)	24 (32%)
UZ Brussels	Belgium	28 (31%)	1 (33%)	0 (0%)	21 (28%)
Total		89	3	4	75

AZ, Algemeen Ziekenhuis; PPT, per-protocol treatment; UZ, Universitair Ziekenhuis.

**Table 2:** Patient demographics

Demographics at baseline	Total (*N* = 89)
Age, years (at enrolment)^a^	62.69 ± 12.26
Gender^b^	*n* = 89
Female	33/89 (37.08%)
Male	56/89 (62.92%)
Height^a^ (cm)	171.63 ± 30.40
Weight^a^ (kg)	79.62 ± 26.49
Systolic blood pressure^a^ (mmHg)	136.41 ± 19.97
Diastolic blood pressure^a^ (mmHg)	79.26 ± 12.13
Heart rate^a^ (b.p.m.)	73.28 ± 22.95
Respiratory rate^a^ (breaths/min)	14.88 ± 5.68
Body temperature^a^ (°C)	36.25 ± 0.43

ECG, electrocardiogram; *n*, counts; *N*, total; SD, standard deviation.

^a^Values are presented as mean ± SD.

^b^Values are presented as *n*/*N* (%).

Prior to the analysis, four subjects were excluded due to procedural errors, where the EGM and electrocardiogram signals were not available. The final patient population for analysis thus included 85 subjects. Among the available EGMs, individual EGMs were excluded from the primary endpoint analysis because of the following reasons: the rhythm was NSR (99 EGMs), inability to confirm the ground-truth isolation labels by the investigator using the predefined criteria (41 EGMs at T-final), and the presence of continuous pacing artefacts (28 EGMs). As a result, the final number of recorded EGMs at T-baseline and T-final available for the analysis was 248 and 250, respectively. In total, the isolation label for comparison with the PVI Analyzer performance could be reliably established for 75 of the 85 patients.

### Ablation procedure

Radiofrequency ablation was performed in 42/85 (49%) patients, and CB ablation was performed in 43/85 (51%). Pulmonary vein isolation was verified at the discretion of the investigators by either a complete abolishment of any PV potentials, observation of automaticity, or confirmation by pacing.

### Primary performance endpoint

The analysis compared 248 baseline (PVI ground-truth-negative) EGMs and 250 final (PVI ground-truth-positive) EGMs (*Table 3*). When evaluated at the prespecified unbiased decision point, the PVI Analyzer correctly classified 215/248 (87%) as not isolated and 216/250 PVs (86%) as isolated during SR. The sensitivity and specificity of the PVI Analyzer during SR were 86 and 87%, respectively, both of which surpassed the prespecified performance criteria (*P* = 0.006 and *P* = 0.004). The positive and negative predictive values of the PVI Analyzer were 216/249 (87%) and 215/249 (86%), respectively, and the diagnostic accuracy of the PVI Analyzer during SR was 431/498 (87%). Evaluated at all isolation index thresholds, the PVI Analyzer classification reached an area under the curve for the receiver operating characteristic (ROC) curve of 92%.

**Table 3:** Classification results for primary endpoint

		PVI Analyzer classification		Total
		Positive (isolated)	Negative (baseline)	
Ground-truth	Positive (isolated)	216 (86.4%)	34 (13.6%)	250
	Negative (baseline)	33 (13.3%)	215 (86.7%)	248
	Total	249	249	498

### Secondary performance endpoints

#### Performance in cryoballoon and radiofrequency subgroups

In the RF subgroup, the PVI Analyzer correctly classified 227 out of 258 EGMs, yielding an accuracy rate of 88% with a sensitivity and specificity of 82 and 94%, respectively, while in the CB subgroup, the PVI Analyzer correctly classified 204 out of 240 EGMs, yielding an accuracy rate of 85% with a sensitivity and specificity of 92 and 79%, respectively (*Table 4*).

**Table 4:** Classification results for radiofrequency and cryo subgroups

Type of procedure	Measurement		PVI Analyzer classification		Total
			Positive (isolated)	Negative (baseline)	
Radiofrequency	Ground-truth	Positive (isolated)	107 (81.7%)	24 (18.3%)	131
		Negative (baseline)	7 (5.5%)	120 (94.5%)	127
		Total	114	144	258
Cryoballoon	Ground-truth	Positive (isolated)	109 (91.6%)	10 (8.4%)	119
		Negative (baseline)	26 (21.5%)	95 (78.5%)	121
		Total	135	105	240

The accuracy rate of the PVI Analyzer in the RF and CB subgroups exceeded the 80% accuracy performance goal (*P* = 0.0007 and *P* = 0.03, respectively).

#### Feasibility of continuous ‘real-time’ PVI Analyzer analysis during pulmonary vein isolation ablation treatment

The estimate of agreement accuracy between the two non-overlapping EGM beats was 96%, exceeding the performance endpoint of 90% (*P* < 0.0001).

#### Performance assessment of PVI Analyzer classification accuracy at the time of expert-defined isolation before the end of the pulmonary vein isolation ablation procedure

During processing, 17 (14.7%) EGMs were rejected by the PVI Analyzer. Of the remaining 99 EGMs, the PVI Analyzer correctly classified 86/99 EGMs, yielding a sensitivity of 87%. The performance exceeded the endpoint criteria of 80% (*P* = 0.04).

#### Comparison of algorithm performance on same data recorded by the ECGenius and conventional electrophysiology systems

Data were extracted from 312 paired EGMs (156 baselines and 156 isolated EGMs) from the ECGenius and conventional systems. Within the paired EGM data, the PVI Analyzer yielded a sensitivity and specificity of 69 and 80%, respectively, using data from the conventional recording system, and a sensitivity and specificity of 84 and 86%, respectively, using data from the ECGenius System. The overall accuracy of the classification based on the ECGenius System data exceeded that of the conventional system (*P* = 0.002).

A paired McNemar’s test, with an *α*-value of 0.05, was applied to analyse the differences in classification performance. The *P*-value when running the test was <0.0001. Using the standard *α*-value of 0.05, we can reject the null hypothesis and conclude that the proportion of errors is, in fact, different for the two data sets.

#### Per vein subgroup analysis

A subgroup analysis showed, as may be expected, that the specificity was lower in the right veins (due to difficulty in CMC positioning in those veins) and the sensitivity was lower in the left veins (due to more far-field activity). However, the study was not powered for such an analysis, and the results did not achieve statistical significance.

#### Algorithm performance in atrial fibrillation

Insufficient data were collected to assess algorithm performance in AF.

## Discussion

### Main findings

This paper presents the results of a study to validate an automated PVI algorithm employing a trained and tested ML classifier to identify isolated veins. The predefined success criteria for the study were a sensitivity and specificity >80% when compared with expert opinion on vein isolation status during the procedure. The algorithm was able to achieve a sensitivity and specificity of 86 and 87%, respectively, with an overall rate of accuracy of 87%.

*Post hoc* analyses of the CB and RF subgroups also showed that the algorithm exceeded the predefined success criteria for accuracy with both modalities and that it was highly sensitive in terms of classification at the time of isolation. Although the RF and CB accuracy rates were not statistically different, the rate of CB accuracy was 79%. This was not a study endpoint but supports the understanding that an initial optimal placement of the CMC that is sensitive to the local vein potentials and not too distal in the vein may, in some situations, be challenging while securing a good balloon occlusion. This may, in some situations, result in suboptimal EGMs that may impact the specificity of the baseline confirmation of non-isolation. Indeed, continuous real-time monitoring of PVI during ablation could be challenging in CB procedures due to CMC positioning within the veins. This is related to the Achieve catheter having a dual role of guide and support wire for the catheter and mapping catheter, and this creates challenges for the operator to satisfy those two needs simultaneously. However, the algorithm exceeded the prespecified performance goal for the secondary endpoints of accuracy of both cryo and RF. The investigators believe that, in spite of the clear differences in the two approaches, the algorithm is robust enough to perform in both situations.

### Disagreement between algorithm and operator

While the study was not specifically designed to assess the reasons for disagreement between the algorithm and the operator, these reasons have been examined, and the results are summarized as follows: the algorithm produced 33 false positives, all of which occurred at baseline. Of these 33 occurrences, 27 have been judged to be due to a distal CMC placement within the PV that recorded low-amplitude, low-frequency far-field EGMs; four EGMs had an element of noise artefact obscuring near-field potentials. The algorithm decision for the remaining two false-positive disagreements cannot be explained. As these false positives were all recorded at baseline in a cohort of patients undergoing a *de novo* PVI procedure, it may be assumed that all veins were un-isolated. In clinical use, an isolated output from the algorithm in this scenario may encourage the operator to place the CMC in a position that yields better PV potentials.

There were also 34 false negatives. After an individual analysis of EGMs, it was found that 5 EGMs had excessive noise; 2 were assumed to be incorrectly labelled on the data collection form; 22 EGMs had sharp elements within the far-field signal; and in 5 disagreements, the algorithm output could not be explained.

Sensitivity (measuring the algorithm’s ability to correctly identify isolated veins) and specificity (measuring the algorithm’s ability to correctly identify non-isolated veins) are regarded as equally important in the algorithm and in the study (hence, the threshold of 50 in the isolation index cut-off).

The consequence of low sensitivity would be a lot of false positives, which could lead to veins not being isolated during a procedure. The consequence of low specificity would be a lot of false negatives, which could lead to veins being ablated more than necessary and a prolonged procedure time. Both consequences are rated equally severe in the algorithm and in the study.

### Importance of assessing the isolated status of the pulmonary vein during radiofrequency and cryoballoon ablation strategies

Creating a durable PVI is essential to reduce the risk of recurrence of AF.^13^ However, in spite of the introduction of new energy modalities, ablation techniques, and catheters, the rate of durability of the veins 180 days after the procedure remains around 85%, as shown in a series of remapping studies.^14–17^ While this is thought to be a result of energy source, tissue contact, or anatomical constraints,^18^ incorrect classification of vein isolation status may play a role in these durability results. The few studies of operator accuracy and inter-operator variability in vein classification that have been performed show less than perfect results.^10,11^ Due to the previously described complexities of delineating near- and far-field potentials, this is hardly surprising, and while pacing manoeuvres are helpful in separating these potentials, this may not always be possible. Single-shot devices are designed to simplify PVI procedures by having a single catheter in the LA. However, this limits the option to pace from the left atrial appendage, which is often the source of far-field potentials, making correct classification difficult. Pacing the distal coronary sinus catheter may be helpful in this case, but not always conclusive.^19^ In addition, the algorithm is agnostic to which of the four PVs are analysed, i.e. the same model is applied to all veins, and it does not have access to information about which vein is currently being analysed.

Improvements in signal processing techniques, coupled with ML, may provide additional information required to increase the accuracy and speed of vein classification. A recent study from a centre in Switzerland showed the results of an algorithm designed to discriminate between near- and far-field EGMs in PV signals collected during CB ablation cases from an eight-pole CMC.^10^ While the authors reported an accuracy rate of 82.7% (specificity 89%, sensitivity 77%) from only two features (low-frequency power and maximum amplitude), they acknowledged that the study was small, analysing only 335 EGMs and based on results from a single centre and ablation modality. This compares to an accuracy rate of 87% for the PVI Analyzer in this multi-centre study.

### Third-generation electrophysiology recording systems improve the performance of the PVI Analyzer algorithm

**Figure 3:**
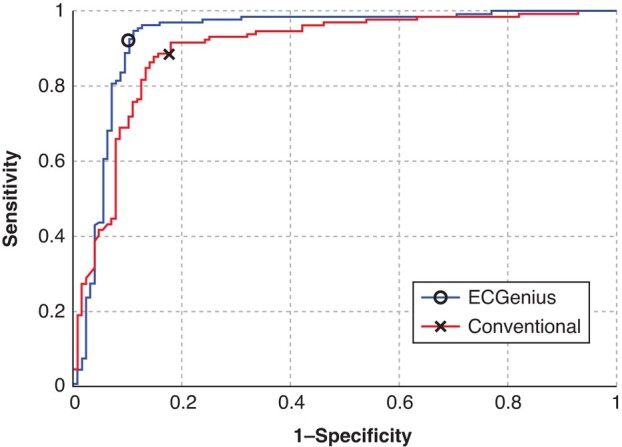
A receiver operator characteristic curve showing the performance of the PVI Analyzer when trained with data from a legacy recording system vs. ECGenius. PVI, pulmonary vein isolation.

The PVI Analyzer™ (CathVision ApS) was originally trained and tested on bipolar EGMs collected from legacy EP recording systems subject to filtering and processing constraints. A recent study comparing the performance of the PVI Analyzer when trained on both unipolar and bipolar signals recorded with a third-generation EP recording system (ECGenius™, CathVision ApS) with higher-quality, unfiltered signals sampled at a higher rate, showed an accuracy improvement rate from 86 to 91% (*P* < 0.05). In comparing the classification performance on paired EGMs, the model trained with ECGenius System data showed superior or equal classification performance at all decision thresholds, as evident in the ROC curve (see *Figure 3*).^20^

The visualization of PV potentials during the delivery of both RF and cryoenergy offers the opportunity to observe a temporal splitting of near- and far-field signals due to delayed activation into the vein. However, with CB placement of the CMC deeper into the vein to help support the balloon catheter position may mean that no PVs are visible. Indeed, the lower-specificity performance of 79% for the CB group, which was attributed to the lack of PV potentials, may have led to an isolated classification by the algorithm. However, the more clinically relevant performance in the setting of *de novo* PVI related to sensitivity, and the algorithm achieved 92% success in classifying isolated veins in CB procedures.

It should also be noted that a CMC position too close to the balloon may lead to noise on the signals due to the insulating effect of the cryo ice ball. A recent retrospective study has shown the importance of visualization of PV potentials during cryoenergy delivery. Veins where PV potentials were not visible during energy delivery were associated with increased AF recurrence (hazard ratio = 1.275; 95% CI 1.134–1.433; *P* < 0.01).^20^ While the PVI Analyzer may assist in cases where PV potentials are captured, further improvements in adaptive and predictive filtering techniques to remove noise from signals may be beneficial.^21^

### Machine learning models

The application of artificial intelligence and ML in medicine in general and in cardiology in particular is growing.^22^ In EP, ML algorithms have been used to predict the likelihood of developing AF,^23^ as well as for the prediction of clinical outcomes after PVI.^24,25^ There are a number of options when considering an ML classifier algorithm, which generally fall into the following categories: linear classifiers, such as linear and logistic regression; tree-based classifiers, for example, decision trees and random forest; neural networks; Bayesian approaches; instance-based classifiers, such as *k*-nearest neighbours; and SVMs. Each technique has its own characteristics, weaknesses, and advantages. The SVM approach has the advantage of being able to handle both linear and non-linear data using kernel functions and differs from logistic regression in that the algorithm seeks to create a hyperplane between outcomes with the highest separation, making it well suited to medical decision-making.^26^ Indeed, the recent study mentioned earlier to classify near- and far-field EGMs in cryoablation PVI procedures compared the outputs of various models such as decision tree, linear discriminant analysis, *k*-nearest neighbours, and SVM and found SVM to have the greatest accuracy.^11^ The PVI Analyzer algorithm was trained using four EGM features from each potential: morphology type; peak-to-peak amplitude; maximal d*v*/d*t*; and sharpest peak angle.^10^ The results of the PVISION study confirm that the SVM technique is sufficiently robust for this type of signal classification.

### Limitations

The PVISION trial was designed as a ‘real-world’ validation of a previously trained and tested ML model to test the agreement between algorithm and physician. It should be noted that such a model may have the greatest utility when the operator is unsure of PVI status, and the study design did not allow for the exploration of why disagreement may have occurred and thus for the establishment of a ‘ground-truth’ PV status. While a previous study has shown the algorithm to perform better than experienced operators in classifying PVI, further studies are required for examining disagreement.

The PVI Analyzer algorithm provides an instantaneous evaluation of vein isolation status based on four EGM features. It is agnostic to catheter position and does not compare pre- and post-ablation EGMs for either morphology or timing and therefore could be sensitive to PV entrance delay that places the EGM within the time window of ventricular activation. The algorithm, therefore, will act as a decision support tool and work with the signals from the catheter at a certain position (antral vs. PV). If the algorithm classifies the vein as isolated before therapy is delivered, it may encourage the operator to consider changing the position of the CMC to visualize PV potentials. In the case of an isolation decision, it may be expected that the operator continues to use their best judgement to confirm the status, including optimizing the quality of the EGM or repositioning the CMC catheter.

PVISION was a multi-centre validation study of an ML algorithm trained and tested for the classification of PV status during cryo or RF PVI studies. Since the completion of the study, a new ablation modality employing a single catheter to map and ablate with pulsed field energy has been introduced and appears to have advantages in terms of procedure efficiency and safety.^17^ The PVI Analyzer algorithm’s performance with this particular modality has been tested only in a limited number of patients^27^ and, due to the nature of the widely spaced, large electrodes, may require further training of the model.

It should also be noted that PVISION allowed data from the eight-pole Achieve catheter and a 10-pole CMC. Signals from other CMCs with differing electrode configurations and signals from multi-spline catheters such as the Pentaray and Octaray (Biosense Webster), which have smaller electrodes,^28^ were not tested, and the performance of the algorithm with these catheters requires further study.

Patients recruited into the PVISION trial were referred for a first-time PVI for paroxysmal or persistent AF. However, most patients were paroxysmal and presented and remained in SR throughout the procedure. The PVI Analyzer has, therefore, not been validated in AF rhythm or for re-do procedures, and further studies are required to substantiate its performance in these settings.

## Conclusions

The PVISION study validated the real-world performance of a previously trained and tested ML algorithm designed to classify PV potentials as either isolated or non-isolated. The study met its endpoint, with the overall agreement of the output being 87% when compared with expert clinical opinion. Considering the difficulty of correctly classifying PV status during PVI procedures, the PVI Analyzer may prove to be a useful tool to confirm PVI during ablation procedures. However, further studies are required to test the performance of the algorithm in AF and with other catheter and ablation modalities.

## Data Availability

The data underlying this article will be shared on reasonable request to the corresponding author.
